# Enhancing histopathological image classification of invasive ductal carcinoma using hybrid harmonization techniques

**DOI:** 10.1038/s41598-023-46239-0

**Published:** 2023-11-16

**Authors:** Nassib Abdallah, Jean-Marie Marion, Clovis Tauber, Thomas Carlier, Mathieu Hatt, Pierre Chauvet

**Affiliations:** 1 LaTIM, INSERM, Université de Bretagne-Occidentale, Brest, France; 2 LARIS, Université d’Angers, Angers, France; 3 Catholic University of the West, Angers, France; 4 Imaging & Brain, Université de Tours, Tours, France; 5 University Hospital of Nantes, Nantes, France

**Keywords:** Breast cancer, Cancer imaging

## Abstract

This study aims to develop a robust pipeline for classifying invasive ductal carcinomas and benign tumors in histopathological images, addressing variability within and between centers. We specifically tackle the challenge of detecting atypical data and variability between common clusters within the same database. Our feature engineering-based pipeline comprises a feature extraction step, followed by multiple harmonization techniques to rectify intra- and inter-center batch effects resulting from image acquisition variability and diverse patient clinical characteristics. These harmonization steps facilitate the construction of more robust and efficient models. We assess the proposed pipeline’s performance on two public breast cancer databases, BreaKHIS and IDCDB, utilizing recall, precision, and accuracy metrics. Our pipeline outperforms recent models, achieving 90-95% accuracy in classifying benign and malignant tumors. We demonstrate the advantage of harmonization for classifying patches from different databases. Our top model scored 94.7% for IDCDB and 95.2% for BreaKHis, surpassing existing feature engineering-based models (92.1% for IDCDB and 87.7% for BreaKHIS) and attaining comparable performance to deep learning models. The proposed feature-engineering-based pipeline effectively classifies malignant and benign tumors while addressing variability within and between centers through the incorporation of various harmonization techniques. Our findings reveal that harmonizing variabilities between patches from different batches directly impacts the learning and testing performance of classification models. This pipeline has the potential to enhance breast cancer diagnosis and treatment and may be applicable to other diseases.

## Introduction

One of the major challenges in biomedical research lies in the necessity to have substantial volumes of patient data to train classification models effectively. However, in the vast majority of biomedical applications, such extensive datasets are not readily available. Consequently, we often resort to pooling patient data from multiple acquisition centers. This practice introduces what is commonly known as the “batch effect,” an artifact attributed to differences in acquisition hardware or protocols. Such batch effects hinder the generalizability of our models. Data harmonization is becoming an increasingly crucial issue in biomedical research. This is done by estimating the batch effect between different centers and minimizing its impact, thereby enhancing the generalizability of the models. This technique has been extensively applied in medical imaging, in FDG PET/CT imaging, as seen in works like^1^ and^2^ and in MRI imaging^3^. However, harmonization of features in histopathological slices is less widespread. Harmonization constitutes a critical, yet intricate, facet of histopathological image classification. Specifically, two principal types of variability serve as obstacles to the robust performance of machine learning algorithms: intra-database and inter-database. Intra-database variability arises from inconsistencies present within a single data collection center, often taking the form of variations in staining or fluctuations in quality across patches within an individual histopathological slide.

Figure 5 illustrates examples of patches from a histopathological slide following unsupervised clustering. One cluster comprises images of border regions, while the other encapsulates images of central regions, thereby underscoring the need for intra-slide harmonization to mitigate such variabilities. Additionally, this intra-slide variability is manifest not only between different cluster patches but also within the same cluster patches across various classes, as illustrated in Fig. 6.

Inter-database variability exists across multiple centers and originates from heterogeneous imaging technologies or diverse acquisition protocols. These variabilities compromise the fidelity of machine learning models, rendering them less reliable and poorly generalizable.

The significance of this research lies in its twofold contribution to histopathological image classification. Firstly, by addressing both intra-database and inter-database variabilities, our approach improves the generalizability and robustness of machine learning models across diverse imaging protocols. This directly contributes to increase diagnostic accuracy. Secondly, the proposed harmonization techniques enhance model reliability, particularly in multi-center clinical settings, thereby impacting early cancer diagnosis and treatment.

To surmount these challenges, our research proposes a harmonization-centric pipeline operational on dual fronts: intra-database and inter-database.

Breast cancer continues to pose a critical public health challenge globally. Early detection remains crucial for favorable patient outcomes but is frequently impeded by the excessive workload and the potential for human error in conventional diagnostic procedures^4^. Artificial Intelligence (AI) and machine/deep learning (ML/DL) have emerged as potent adjuncts to human expertise in clinical diagnosis, and in certain scenarios, surpass it^5^.

The focal point of our research is to bridge the existing research gap by concentrating on both intra-database and inter-database harmonization methods. Within the domain of intra-database harmonization, we introduce methodologies to standardize patches within each histopathological slide, thereby alleviating intra-slide variability and augmenting classification performance. For inter-database harmonization, we implement techniques to synchronize data across disparate databases, thereby yielding a consolidated and robust training set.

Several preceding studies have ventured into data harmonization in the context of medical imaging. For instance, the ComBat technique, developed by Johnson et al., aimed to ameliorate non-biological variations often found in microarray data^6^. Subsequent adaptations of this method extended its application to harmonize data in PET/CT/MRI imaging^7,8,9,10,11^. However, these works have largely focused on inter-database harmonization, neglecting the challenges associated with intra-database variability.

To address this research gap, our study employs a pipeline that melds feature engineering with harmonization techniques. Specifically, we propose a novel strategy for harmonizing patches categorized as atypical, as well as clusters produced through unsupervised classification techniques. In doing so, we aspire to enhance the reliability and accuracy of histopathology classification models.

The pipeline undergoes evaluation in the setting of classifying histopathological slides as either cancerous or non-cancerous, using data from two publicly accessible databases. Our objective entails assessing multiple harmonization methods to navigate both intra- and inter-database variabilities, thereby facilitating the selection of the most suitable model for accurate classification. Our contributions extend beyond mere classification tasks. We introduce a robust, harmonization-focused methodology aimed at bolstering the reliability and generalizability of machine learning models employed in histopathological image classification, thus catalyzing advancements in early cancer detection and treatment.

## Materials and methods

Benign histology refers to a tumor that does not meet any criteria for malignancy, is growing slowly and is well localized. On the contrary, malignant tumors are synonymous with cancer: the lesion may invade and destroy adjacent structures (locally invasive) and expand to distant organs (metastatic). Benign and malignant breast tumors can be classified into different types based on the appearance of the tumor cells under the microscope. Different types/subtypes of breast tumors may have different prognosis and therapeutic implications. In the present work, we focused on the classification of benign and malignant types of breast cancer, particularly invasive ductal carcinoma (IDC), which is a common subtype of malignant breast tumor.

### Dataset

We used two publicly available datasets of histopathological images of breast tumors for our study: the Invasive Ductal Carcinoma (IDC) dataset^12^ and the Breast Cancer Histopathology Image Classification (BreakHis) dataset^13^. Both datasets contain digitized images of histopathological slides, and have been used extensively in previous research on breast tumor classification using machine learning techniques.

The IDC dataset includes images of invasive ductal carcinoma from 162 patients, scanned at 40x magnification with a whole slide scanner. A total of 277,524 patches of size 50x50 pixels were extracted from these slides, of which 78,786 were positive for IDC and 198,738 were negative. The dataset was annotated using Aperio’s ImageScope visualization software. This dataset was chosen for its large size and well-defined target variable (IDC vs. non-IDC).

The BreakHis dataset, on the other hand, contains images of both benign and malignant breast tumors of different histological types. It includes 9,109 microscopic images of breast tumor tissue collected from 82 patients at different magnification factors. This dataset was built in collaboration with the P &D Laboratory - Pathological Anatomy and Cytopathology, Parana, Brazil. It currently contains four distinct histological types of benign breast tumors and four malignant tumors. This dataset was chosen for its diversity of histological types, which can have different implications for prognosis and treatment.

Both datasets present potential intra and inter variabilities due to differences in acquisition parameters, scanner type, and staining protocols, among others (Table 1). These variabilities can affect the performance of machine learning models trained on these datasets, and thus highlight the need for harmonization techniques to reduce their impact. In the following sections, we describe the harmonization methods used in our study to address these variabilities.

**Table 1:** Information and distribution of images on IDC and BreaKHis databases.

Dataset	Magnification	Source	Acquisition material	Benign	Malignant
IDC-DB	40 X	US	Scanner ($0,25\mu m$/ pixel) + Aperio ImageScope	198 738	78 786
BreaKHis	40 X	Brazil	Microscope Olympus BX-50 + Samsung digital camera SCC-131AN	652	1370
BreaKHis	100 X	Brazil	Microscope Olympus BX-50 + Samsung digital camera SCC-131AN	644	1437
BreaKHis	200 X	Brazil	Microscope Olympus BX-50 + Samsung digital camera SCC-131AN	623	1390
BreaKHis	400 X	Brazil	Microscope Olympus BX-50 + Samsung digital camera SCC-131AN	588	1232

## Methods

**Figure 1:**
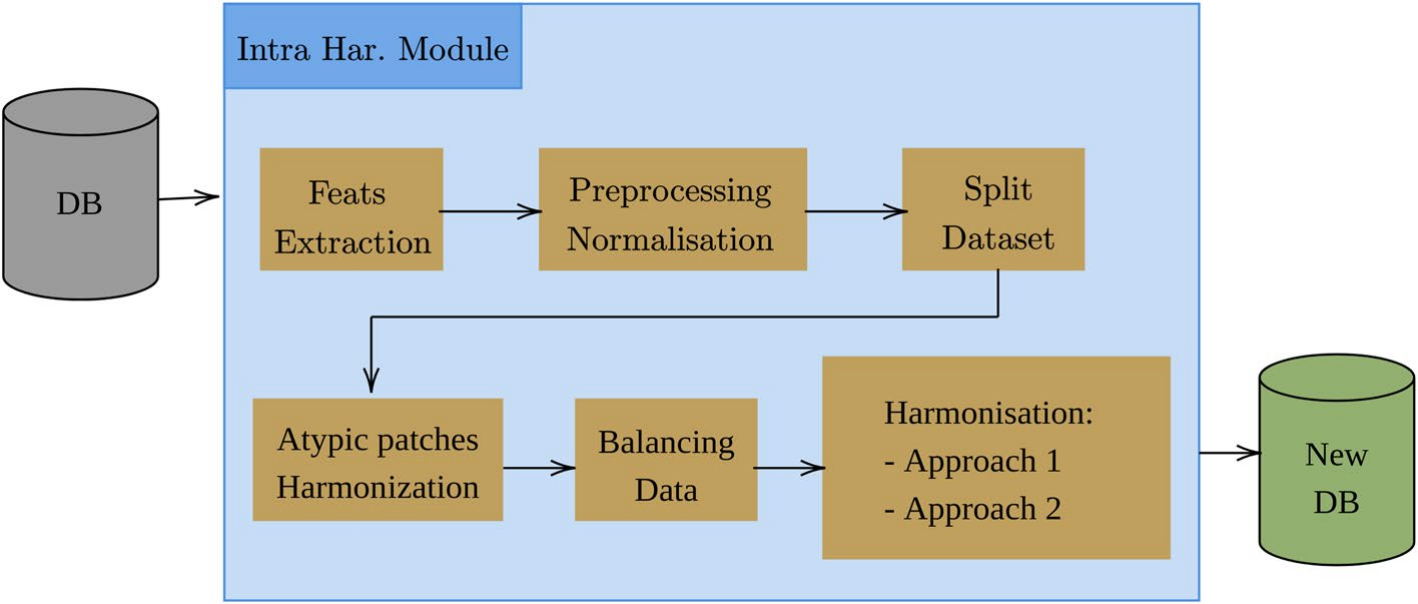
The architecture of our intra-base harmonization module, consisting of 6 steps. The input is a database; the first step is the extraction of features, followed by a normalization of the different groups of features. Then, a split into learning and testing is performed, followed by a processing on the learning samples to reduce the intra-base variabilities.

**Figure 2:**
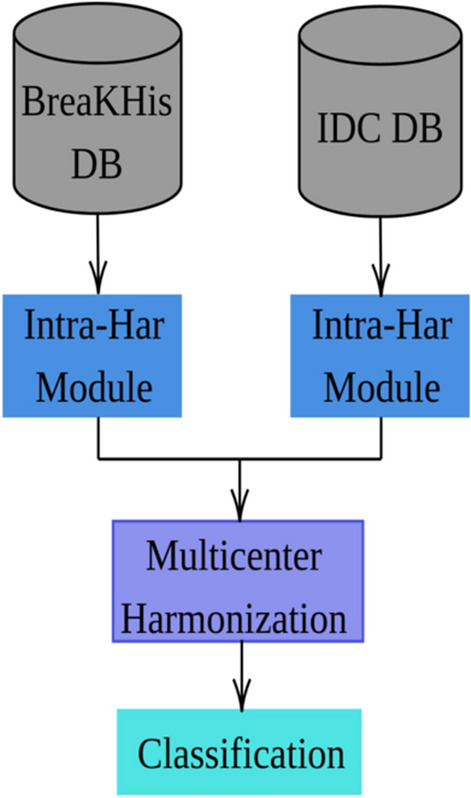
Our complete pipeline: the first step consists in applying the intra-database harmonization module to each database. The second step consists in applying the inter-database harmonization module to the data from different sources (here the two databases). The last step consists in training the classifier.

**Figure 3:**
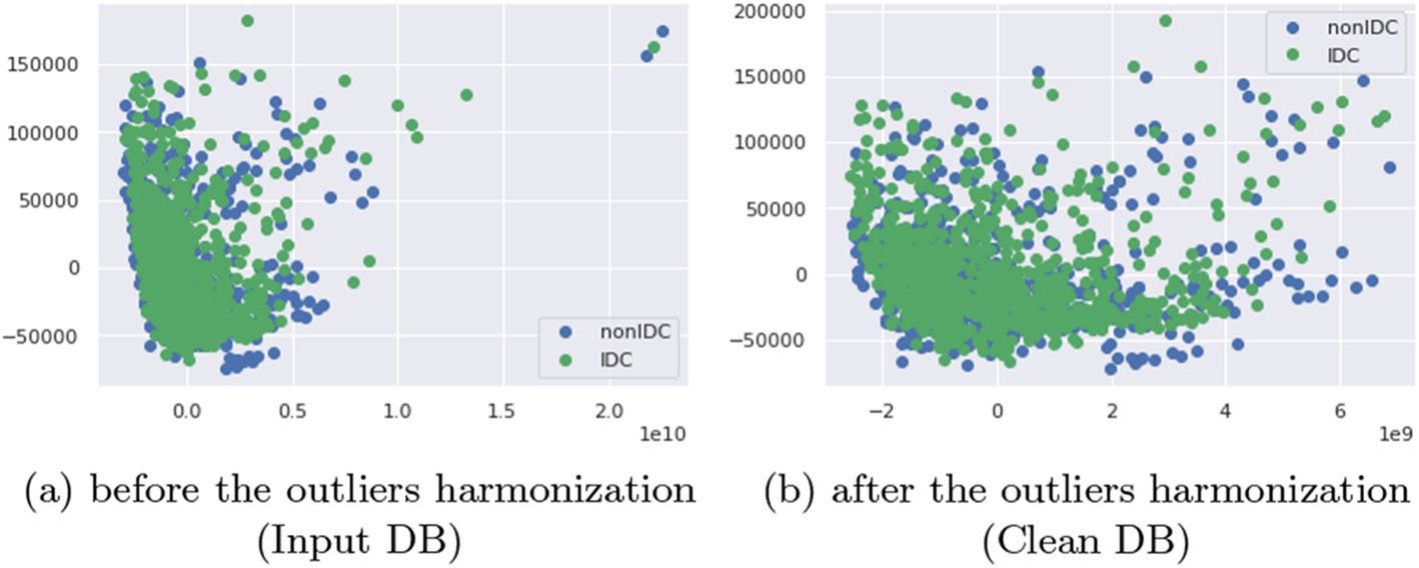
The projection of the samples onto the principal factorial plane, both before and after harmonization, elucidates the impact of our methodology on the projected scatterplot. As illustrated, patches with either IDC or non-IDC subtypes can exist as outliers within the entire dataset and need to be aligned closer to the reference scatterplot, which comprises the majority of samples.

We delineate the technical specifics of the pipeline we engineered for the task of histopathological image classification, with a particular emphasis on its modular architecture. We have incorporated two harmonization modules to tackle the complexities arising from intra-database and inter-database variabilities. The intra-database harmonization module (depicted in Fig. 1) incorporates a series of pre-processing blocks designed to minimize within-database disparities. These blocks are optimized to handle anomalies that cannot be classified as outliers but still demonstrate distinct characteristics, thus potentially biasing model training (see Fig. 3). Indeed, in the proposed methodology, we have designed a pipeline that extracts radiomic features. Following normalization, the data are partitioned into training and test sets (adhering to the literature percentages for both training and testing sets). Subsequent processing is applied exclusively to the training set; however, these procedures are extrapolated to the test data without including them in the training process. For the objective of multi-center adaptability, our pipeline features an additional harmonization module. This system, illustrated in Fig. 2, serves to improve the model’s resilience when exposed to data from different centers.

The pipeline was implemented using Python 3.7 with the TensorFlow library and Keras in their versions 2.3.0. We evaluated the different models using balanced accuracy, recall, and precision as metrics.

In the proposed methodology, we designed a pipeline that extracts radiomic features. Following normalization, the data is partitioned into training and test sets. Subsequent treatments are applied to the training set, while the derived insights are extrapolated to the test set without incorporating them into the learning process..

### Features extraction

For feature extraction, we employed the classical four groups of features: histogram, textural, entropy, and moments, which are described below.

#### Histogram features

The colors of histopathological sections play a crucial role in the detection of cancer cells. Hematoxylin-eosin (HE) is a coloring substance commonly used in clinical practice to bind to cancer cells and make them more visible under a microscope^14^. In this context, we constructed histograms from the red, green, and blue channels and computed histogram features, as listed in Table 2.

#### Textural features

The Gray Level Co-occurrence Matrix (GLCM) is a texture characterization method that considers the spatial relationships of pixels in an image. The GLCM characterizes texture based on the frequency of occurrence of pixel pairs with specific values and a specified spatial relationship. We calculated four GLCM features (correlation, homogeneity, energy, and contrast) on each RGB color.

#### Entropy features

Entropy is a statistical measure of the information content of a signal or image. Various forms of entropy^15^ are adapted to specific cases of study, such as images or signals. We used the sample entropy^16^ on the signal representing the values of the histogram and calculated simple entropy and Shannon entropy on the R, G, and B images separately. We then concatenated them to form a vector representing all the entropies of the image.

#### Moments features

We included moment features in our pipeline that represent the weighted average of image pixel intensities. To do this, we computed statistical moments and Hu moments on the R, G, and B images separately. We then concatenated these different moments to form a new feature vector for each image.

**Table 2:** Summary of the extracted features.

Feature group	Features
Histogram (R,G,B)	Absolute energy, sum over the absolute value of consecutive changes, benford_correlation, count above mean, count_below_mean, first_location_of_maximum, first_location_of_minimum, cid_ce, minimum, maximum, median, kurtosis, longest_strike_above_mean, longest_strike_below_mean, mean_abs_change, mean_change, mean_second_derivative_central, variance, variance_coefficient, percentage_of_reoccurring_datapoints_to_all_datapoints, percentage_of_reoccurring_values_to_all_values, skewness, ratio_value_number_to_series_length, standard_deviation, sum_of_reoccurring_data_points,sum_of_reoccurring_values, sum_values, variance
Texture	Correlation, homogeneity, energy, contrast
Entropy	Shanon entropy, simple entropy, sample entropy
Moment	Moments, hu moments

### Preprocessing and normalization

As we calculated different groups of features, including histograms, GLCM, and entropies, we considered two methods to normalize the data: StandardScaling and RobustScaling. In both methods, the Not a Numbers (NaNs) were treated as null values. However, we opted for the RobustScaling method because it is robust to outliers, unlike the classical StandardScaling method, which we later coupled with the ComBat harmonization. the scaling follows the formula (1):

1 $$\begin{aligned} scaled\_value = \frac{\bigl (value - median\bigr )}{ \bigl (IQR\bigr )} .\end{aligned}$$

The RobustScaling method removes the median and scales the data to the interquartile range (IQR), which is the difference between the 1st and 3rd quartiles. The centering and scaling of this method are not influenced by the presence of a number of marginal outliers based on percentiles. We implemented this method using the Python library “sklearn”.

To ensure comparability with previously published results, we split the datasets according to the previously used ratio. The train and test datasets were stratified based on the target variable.

### Intra-database harmonization module

This section aims to identify patches within the training sets of both IDCDB and BreaKHIS that are considered outliers or atypical. The objective is to bring these samples into closer proximity, thereby reducing the variance between patches containing similar information. For data visualization, we applied Principal Component Analysis (PCA)^17^ to the standardized data using StandardScaling. We then projected the data onto the first two factorial axes to visualize the results obtained (see Fig. 3). We assumed that the distant points (atypical data) in the scattered plot are patches from a different source than the main point cloud. The PCA plot revealed the presence of many outliers.

In our study, we utilized a dataset comprising pre-segmented histopathological patches. Among these, patches located at the borders contain essential information about the tumors. We categorized these border patches as “atypical” for the purpose of analysis. Our aim was to classify these atypical patches using various outlier detection techniques. To achieve this, we evaluated the performance of several established algorithms, such as Isolation Forest^18^, Local Outlier Factor (LOF)^19^, Elliptic Envelope, and One-Class Support Vector Machine (One-Class SVM)^20^. Each algorithm generated a binary mask, classifying each sample as either atypical or non-atypical.

**Figure 4:**
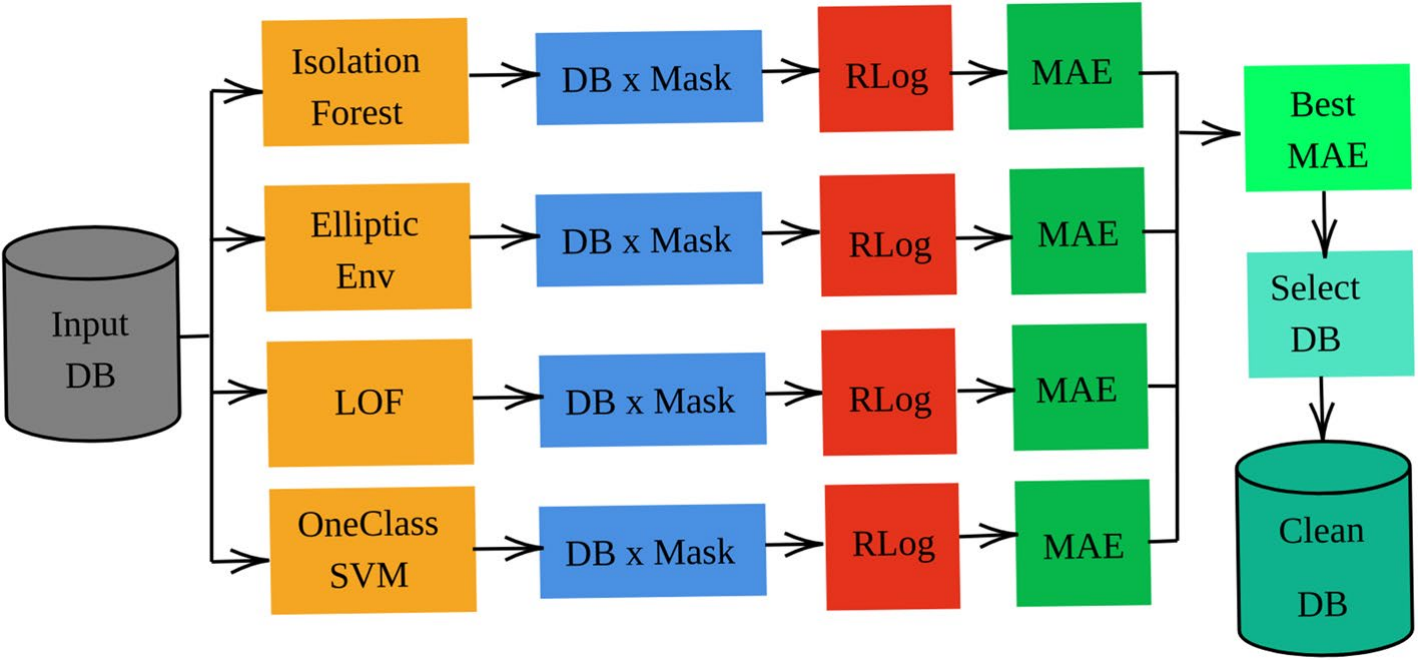
Flow diagram for outliers detection: the first step consists in applying the outlier’s detection methods. Based on the results, the second step consists in classifying the samples as atypical or normal. The third step consists in training a logistic regression model to classify IDC/nonIDC patches on the atypical-free datasets. Finally, the last step consists in selecting the best model based on the MSE criterion (the classification performance of the RLog model).

To scrutinize these categorizations further, we trained a logistic regression model using the generated binary masks as target labels. The effectiveness of each outlier detection method was determined based on the Mean Squared Error (MSE) evaluation metric. This methodology is illustrated in Fig. 4.

**Figure 5:**
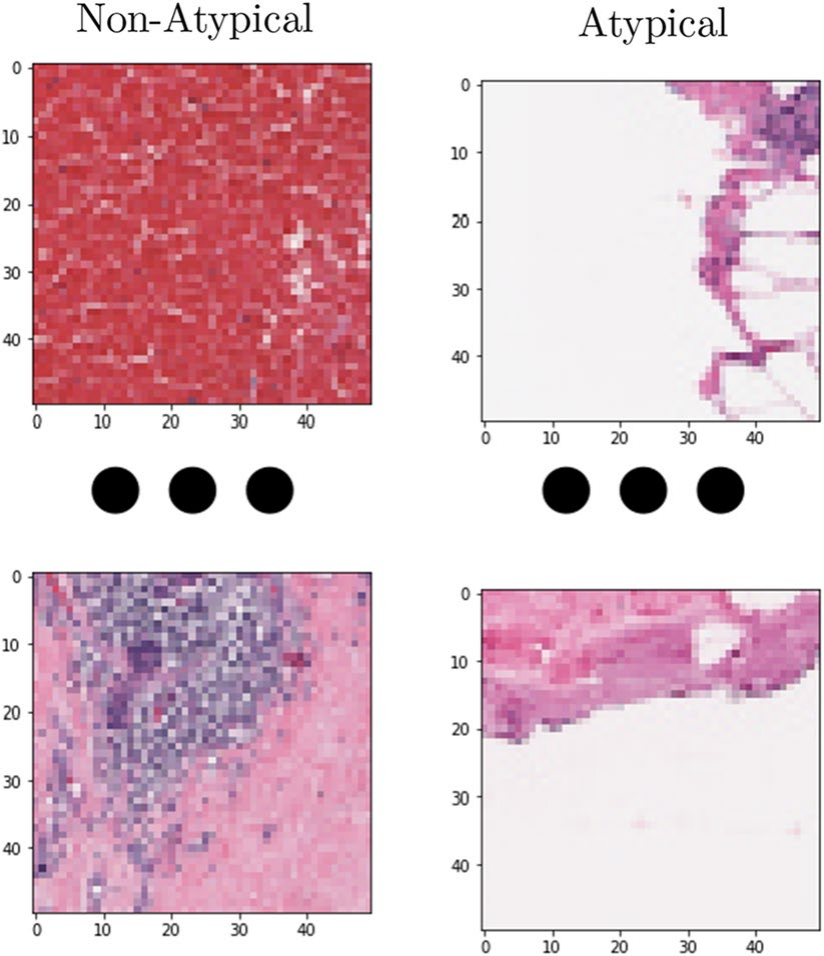
On the left, we present examples of central patches, which constitute the majority within the entire histopathological slide. On the right, we showcase examples of border patches. These two distinct types of patches are invariably present in histopathological studies, as they result from the segmentation of a whole slide.

Upon identifying the most efficient outlier detection algorithm, we applied it to the entire dataset. The dataset was then divided into two subsets: one containing atypical patches (border patches) and another consisting of non-atypical patches (primarily central patches from the histopathological slides). The results of this classification are presented in Fig. 5.

To harmonize the features extracted from the atypical and non-atypical patches, we used the ComBat method^6^. This method is designed to eliminate non-biological variability or “batch effects” that commonly occur in multiple batches of microarray experiments. The ComBat method estimates the parameters that represent batch effects using Empirical Bayesian (EB) techniques^6^. The ComBat method has been widely used in the biomedical fields to harmonize multicenter data^7,8,10,21^. The model equation follows formula (2):

2 $$\begin{aligned} Y_{ijg} = \alpha _g + X\beta _g + \gamma _{ig} + \delta _{ig}\varepsilon _{ijg}, \end{aligned}$$

where $Y_{ijg}$ represents the expression value for the patch *g* for sample *j* from batch *i*. *X* is the design matrix for sample conditions, multiplied by the $\beta _g$ that contains the regression coefficients corresponding to the design matrix. $\gamma _{ig}$ and $\delta _{ig}$ are considered as the site parameters (additive and multiplicative batch effect). Thus, they are estimated using EB and removed in the final adjustments.

The harmonization of the data was done on the main point cloud and on the atypical data using a batch vector containing logic values (01 for a patch belonging to the point cloud and 10 for a patch belonging to the atypical patch cluster).

The harmonization process involved four steps. Firstly, we created a design matrix containing the covariates of the patches and the batch values. Secondly, we standardized the data across features to prepare it for fitting. Thirdly, we fit a linear model to the data to compute the $\gamma ^{*}$ and $\beta ^{*}$ parameters that represent the batch effect. Finally, we adjusted the data by removing the computed batch effect, resulting in harmonized features across the entire dataset.

As depicted in Fig. 3b, the intra-base harmonization approach successfully reduced the intra-class distance and brought the outliers closer to the main point cloud, resulting in a significant reduction in the patch-level variability.

**Figure 6:**
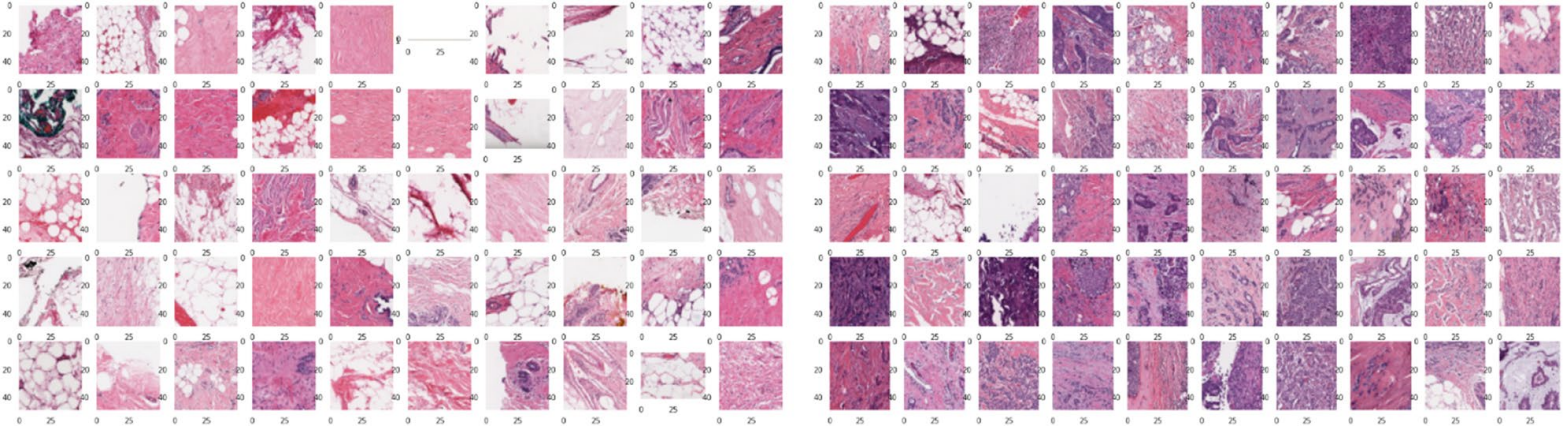
Representation of patches grouped by class: the patches on the right contain no malignant tumor whereas those on the left contain malignant tumor.

We propose an additional harmonization step to reduce the variability between patches. We employed unsupervised learning techniques to generate batch vectors, adapting the index “*i*” in the equation of the ComBat method (Eq. (2)). We compared two approaches: harmonization between cluster patches or between cluster patches within each class (see Fig. 6).

For the first approach, we followed three steps: (1) finding an optimal number of clusters using various methods, such as the silhouette method, the Calinski Harabasz index, and hierarchical agglomerative clustering; (2) generating a batch vector to classify the samples according to the optimal cluster number; and (3) applying harmonization using the ComBat method by considering the variability to be removed in the difference between cluster patches.

In the second approach, we aimed to reduce the intra-class distance and enhance the inter-class distance. We used the same procedure as the patch harmonization presented earlier, but applied separately to the samples of each class. We separated the samples from the different classes, applied patch harmonization to each group, and then regrouped all the samples together. We performed a double harmonization since we have two classes where the *j* of the combat method in the Eq. (2) is either benign or malignant, and the *i* is calculated from the unsupervised clustering within each class, while the *g* is the patch.

### Inter-database harmonization module

Although our pipeline includes harmonization within each database, we also addressed the potential differences between the two databases used in this study. To reduce this variability, we applied the ComBat method. The harmonization process followed the same steps as described in Section "Intra-database harmonization module, which involved standardization by samples, estimation of site parameters, and adjustment of the data by subtracting the estimated parameters.

### Classification

The datasets used in our study are highly imbalanced, with non-cancerous patches being much more prevalent than cancerous ones. Specifically, the IDC dataset contains approximately 60% non-cancerous patches and 40% cancerous patches, while the Breakhis dataset contains 864 non-cancerous patches and 625 cancerous patches.

To address this imbalance, we implemented a synthetic minority oversampling technique (SMOTE)^22^, which generates synthetic samples of the minority class to balance the dataset for training the models. We used the SMOTENN variant^23^, which combines undersampling using the Edited Nearest Neighbors method and oversampling using the SMOTE method and the RandomUnderSampler technique, for quick and easy balancing of the minority class by randomly selecting a subset of the data for the targeted class.

Our pipeline allows for any type of classifier to be used in this step. In our study, we chose to use a multi-layer perceptron (MLP) neural network with a specific architecture presented in Table 3. We used the same model for all tests to enable comparison of outcomes for the different choices implemented in the pipeline. The MLP has two fully connected layers with a “ReLu” activation function. We chose the number of neurons to be equal to the number of features injected into the model, and we handled overfitting using two “dropout” layers at a rate of 0.2. The final layer of the model is a layer with two neurons corresponding to the two classes (malignant/benign) and a “softmax” activation to assign probabilities to each input for belonging to one of the two classes. We applied an elastic net regularization (L1 and L2) on the kernel with weights, bias, and activation function.

We set the number of epochs to 100 and the minibatch to 32. We monitored the loss function on the validation set so that the learning rate (LR) was reduced by a factor of 0.25 when the loss function no longer improved for three successive epochs. If there was no longer an improvement after this LR reduction for three successive epochs, we stopped the training and restored the best weights from the epochs with the best value. We relied on the TensorFlow libraries and functions “Regularizers”, “ReduceLROnPlateau”, and “EarlyStopping” for implementation.

**Table 3:** Architecture of the MLP model.

Layer (type)	Output shape
Dense (Dense)	(277 524, 256)
Activation (Relu)	(277 524, 256)
Dropout (Dropout)	(277 524, 256)
Dense_1 (Dense)	(277 524, 256)
Activation_1 (Relu)	(277 524, 256)
Dropout_1 (Dropout)	(277 524, 256)
Dense_2 (Dense)	(277 524, 2)
Activation_2 (Softmax)	(277 524, 2)

## Results

In this section, we present the most significant results obtained from the different tests we performed. To facilitate the explanation of the results of each model, we assigned a unique identifier to each model, which we specify in the first column of each table. Similarly, we used the character ’M’ to represent malignant tumors (invasive ductal carcinoma) and ’B’ to represent benign tumors in the ’label’ column of Table 4. This table shows all the different model configurations that we tested.

**Table 4:** All the configurations of the models discussed in the results.

ID	Standard scaling	Robust scaling	ComBat for outliers	SMOTENN	Down sampling	Harmonization by patch	Harmonization by patch by class
Configuration 1		x					
Configuration 2	x						
Configuration 3	x		x				
Configuration 4	x				x		
Configuration 5	x			x			
Configuration 6	x		x	x			
Configuration 7	x			x		x	
Configuration 8	x		x	x		x	
Configuration 9	x		x	x			x
Configuration 10	x			x			x

### Base models

Table 5 presents the results of our base models, which did not use any harmonization techniques apart from Standard Scaling.

**Table 5:** Base models for IDC and BreaKHis databases using 70% for training and 30% for testing.

Base model	Training (%)	Validation (%)	Testing (%)
IDC database	83.40	83.47	67.04
BreaKHis database	98.27	88.95	89.61

### RobustScaling vs ComBat outlier harmonization

In the Table 6, we present a comparison between the results obtained between our ComBat-based harmonization method for outliers and the RobustScaling method which also aims at normalizing data taking into consideration the outliers.

**Table 6:** Overview of results from models using different normalization techniques.

Dataset	Configuration model	Training (%)	Validation (%)	Testing (%)
IDC	Configuration 1	89.36	87.48	87.48
IDC	Configuration 2	89.45	87.87	87.58
**IDC**	**Configuration 3**	**87.12**	**86.75**	**87.48**
BreaKHis	Configuration 1	100	92.34	94.40
BreaKHis	Configuration 2	99.27	92.34	93.29
**BreaKHis**	**Configuration 3**	**100**	**93.30**	**95.30**

Significant values are in bold.

### Balancing

In the Table 7, we present the comparative results between the two database balancing methods SMOTENN and DownSampling.

**Table 7:** Overview of results from models using different database balancing techniques.

Dataset	Model configuration	Training (%)	Validation (%)	Testing (%)
IDC	Configuration 4	81.39	80.34	74.88
IDC	Configuration 5	92.74	92.32	79.16
IDC	Configuration 6	95.47	95.03	82.00
BreaKHis	Configuration 5	100	97.47	92.61
BreaKHis	Configuration 6	100	98.46	91.00

### Harmonization ByPatch/ByPatchByClass

In Table 8, we present a comparison between the results obtained by our two approaches of patch harmonization.

**Table 8:** Overview of results from models after ByPatch/ByPatchByClass harmonization: all the presented models use the StandardScaling method for the normalization.

Dataset	Model Configuration	Training (%)	Validation (%)	Testing (%)
IDC	Configuration 6	96.26	95.60	81.00
IDC	Configuration 7	99.95	99.93	76.63
IDC	Configuration 8	95.92	95.29	82.00
IDC	Configuration 9	99.99	99.98	76.00
BreaKHis	Configuration 6	99.61	96.00	89.00
BreaKHis	Configuration 7	100	98.99	81.00
BreaKHis	Configuration 8	100	94.80	90.00
BreaKHis	Configuration 9	100	98.46	81.00

### Best models for IDC and BreaKHis

In order to better detail the performance of these best models that perform better than current state-of-the-art, we provided the recall, precision and F-score values (Tables 10 and 9), along with the different options of each model that led to the results of each.

In Tables 9 and 10, we present the detailed results of the best models obtained for both IDC and BreaKHis databases. Note that the F-score is the harmonic mean of the precision and the recall values.

**Table 9:** Detailed results for the top four models, based on the BreaKHis database, using 20 different splits of 70% for learning and 30% for validating, are presented below.

Model	Options	Label	Precision (%)	Recall (%)	F-score (%)	Testing accuracy (%)
Model_1	Config1	M	89.17	88.26	88.71	90.15 ±3.0
		B	90.91	91.63	91.27	
Model_2	Config3	M	92.89	86.73	89.71	92.15 ±2.2
		B	90.15	94.82	92.42	
Model_3	Config6	M	91.10	93.88	92.46	**93.40±1.8**
		B	95.10	92.83	93.95	
Model_4	Config8	M	86.43	87.75	87.8	88.03±3.7
		B	90.32	89.24	89.78	

Significant values are in bold.

**Table 10:** Detailed results for the top four models, based on the IDC database, using 20 splits partition of 75/25 of the IDC dataset.

Model	Options	Label	Precision (%)	Recall (%)	F-score (%)	Testing accuracy (%)
Model_1	Config1	M	77	88	82	81.608±2
		B	85	74	79	
Model_2	Config3	M	80	86	83	81.726±1.6
		B	85	78	81	
Model_3	Config6	M	83	83	83	83±1.7
		B	82	83	83	
Model_4	Config8	M	93	91	92	**92.73**±**2**
		B	92	95	93	

Significant values are in bold.

### Comparison with existing models

In table 11 and 12, we present the best four models, based on the BreaKHis and IDC databases respectively, that outperform existing models in the literature for model based on features engineering.

**Table 11:** Comparison between our best models and existing work using the same BreaKHis database with the same x40 magnitude of histopathological images and the same ratio for model learning and validation.

Existing work	Ratio Train/Validation	Recognition rate (%)
Spanhol et al. 2015	70/30	83.90
Sanchez-Morillo et al. 2018	70/30	88.32
Boumaraf et al 2021	70/30	87.69
Our work - model 4	70/30	88.00±3.7
Our work - model 1	70/30	90.15±3.0
Our work - model 2	70/30	92.15±2.2
**Our work - model 3**	70/30	**93.40±1.8**

Significant values are in bold.

**Table 12:** Comparison between our best models and existing work using the same IDC database with the same x40 magnitude of histopathological images.

Existing work	Ratio Train/Test	Recognition rate (%)
Celik et al., 2020	80/20	92
Asare et al., 2020	80/20	89.92
Soumya et al., 2021	75/25	92.55
Choudhary et al., 2021	70/30	92.07
model_Config1	75/25	81.608 ±
model_Config2	75/25	81.726 ±.6
model_Config3	75/25	83.12 ±.7
**model_Config4**	75/25	**92.7**±**2**

Significant values are in bold.

### Multicenter models

In Table 13, we present the comparative results among three models that used the multicenter harmonization. The best performing model is MultiC 1, which achieved an accuracy of 95.67% and 95.04% for training and validation, respectively, and 80% and 81% for testing on IDC and BreaKHis databases, respectively. These results demonstrate the effectiveness of multicenter harmonization in improving the robustness of the models against external data.

**Table 13:** Results on the best multicenter models: all presented models use StandardScaling for normalization and SMOTENN for database balancing. MultiC: Multicenter.

Multicenter model	Dataset	Model configuration	Training (%)	Validation	Testing on IDC	Testing on BreaKHis
Model IDC	IDC	2	89.46	87.87	87.55	58.16
Model BreaKHis	BreaKHis	2	99.27	92.34	47.20	93.28
**MultiC 1**	IDC ; BreaKHis	2	**95.67**	**95.04**	**80.00**	**81.00**
**MultiC 3**	IDC ; BreaKHis	3 intra-base	**97.24**	**95.83**	**83.67**	**67.33**
MultiC 4	IDC ; BreaKHis	7	96.03	95.22	74.00	63.00
MultiC 5	IDC ; BreaKHis	9	99.94	99.91	77.00	69.00
MultiC 6	IDC ; BreaKHis	10	99.98	99.94	74.43	67.56

Significant values are in bold.

### Best multicenter models

In Table 14, we present the detailed results of the best multicenter models for the classification of malignant and benign patches.

**Table 14:** Detailed results of the best multicenter models by test databases and labels (M: Malignant, B: Benign).

Model	Test database	Label	Precision (%)	Recall (%)	F-score (%)	Testing Acc. (%)
MultiC Config2	BreaKHis	M	57.9	92.8	71.4	67.3
		B	89.5	47.4	62	
	IDC	M	90.7	86	88.3	83.7
		B	68.9	77.8	73.1	
MultiC Config3	BreaKhis	M	70.9	93.4	80.6	81
		B	93.1	70.1	80	
	IDC	M	95	77.3	85.4	80
		B	61.3	90.2	73	
MultiC Config8	BreaKHis	M	65.9	89.8	76.1	75
		B	89	63.7	74.3	
	IDC	M	95.4	77	85.3	81
		B	61	91	73.9	

## Discussion

Initially, we trained models without any data pre-processing except for normalization to establish a baseline for comparison. These base models achieved a testing balanced accuracy of approximately 67% for the IDC database and almost 90% for the BreaKHis database (Table 5). The disparity in performance can be partially attributed to the larger size and greater diversity of the IDC database (277,524 images) compared to BreaKHis (1,490 images).

Drawing on the baseline models, we compared our proposed approaches for outlier harmonization within each database. We observed very similar performances (Table 6), with a slight advantage for the ComBat outliers harmonization after StandardScaling normalization approach ( 95.3%,  87.48%) over the RobustScaling only approach ( 94.4%,  87.48%) on the BreaKHis and IDC databases, respectively. The results in Table 6 compare the outcomes obtained from our three different configurations: Configuration 1 refers to RobustScaling, Configuration 2 to ComBat harmonization without outlier detection, and Configuration 3 to ComBat harmonization with outlier detection. With the best pipeline options determined (i.e., ComBat outliers harmonization and StandardScaling normalization), we evaluated the techniques implemented to alleviate the balancing problem between malignant and benign patches. The DownSampling method achieved classification scores of 81.39%, 80.34%, and 74.88% on training, validation, and test sets for the IDC database. Conversely, the SMOTENN method outperformed DownSampling, yielding improvements of 10% on the training and validation sets and 5% on the test set for the same database (Table 7).

After balancing the number of patches, we assessed our pipeline’s components designed for per-patch harmonization to reduce variability between patches containing the same information. We selected the best models resulting from the previous stages.

The best outcomes obtained from this stage correspond to the models where we applied StandardScaling normalization, outlier harmonization with ComBat, balancing with the SMOTENN method, and Harmonization between cluster patches (Table 8). These results help identify the best options to implement in our proposed pipeline, achieving high performance in differentiating malignant and benign patches across the two distinct databases. The highest performance was observed in Configuration 8 for the BreaKHis dataset, with 100% accuracy on the training set, 94.80% on the validation set, and 90.00% on the testing set. This suggests that the ByPatch harmonization, combined with the previously selected optimal pipeline options, was most effective in reducing variability between patches and improving classification performance. In contrast, Configuration 6 and Configuration 8 for the IDC dataset yielded comparable results, with Configuration 8 exhibiting slightly better performance on the testing set (82.00% compared to 81.00% for Configuration 6).

### Comparison of our results with existing literature

After selecting the different blocks of the pipeline, we compared the results of our best models with recently published works using the feature engineering approach on the same datasets. In order to make the results comparable, we selected the models using the BreaKHis database with a ratio of 70% for model training and 30% for model testing with a zoom level of histopathological patches at x40 (Table 11). Four of our models obtained higher testing accuracy (90% - 95.3%) than the results published in recent works (ranging between 83.9% and 89.8%) and where the maximum achieved in^24^ were 89.8%. As shows Table 12, Celtik et al. 2020^25^ achieved 91.57% balanced accuracy with their DenseNet-161 model and 90.96% balanced accuracy using the ResNet-50 architecture on IDC data. Soumya et al., 2021^26^ used a set of 782 features computed on the IDC dataset, followed by feature selection using Pearson’s correlation coefficient to obtain a dataset with four features that are then used for classification and yielded the highest accuracy (92.55%). Asare et al. 2020^27^, proposes a simple convolutional neural network model to distinguish benign and malignant breast cancer tumors in histopathological images in the IDC dataset by using different optimization algorithms and implementing several data augmentation techniques that regulate overfitting and improve the generalization ability of the proposed model. The accuracy, sensitivity, and specificity obtained were 89.92%, 94.02%, and 86.42%, respectively. Also, choudhary et al. 2021^28^, based their models on the three popular pre-trained CNNs, VGG19, ResNet34 and ResNet50. They obtain 91.25% with VGG19 while with ResNet34, the accuracy increases slightly to 91.80% and finally the best accuracy was obtained using the ResNet50 model with an accuracy of 92.07%. The results obtained by our models show the importance of using the ComBatOutliers method coupled with the data balancing method. Indeed, with our model having the Config4 configuration, we obtain an average accuracy of 92.7% on 20 splits and a maximum accuracy of 94.7%. The advantage of this approach compared to deep learning, in Refs.^25,27–35^, is the interpretability of our models. Indeed, the different configurations used reveal the requirements of the features in terms of harmonization by a priori defined variability which was in our case the edge images with the central images, balancing between the numbers of malignant and benign patches and harmonization of atypical data within the database for a better classification. Moreover, as with traditional neural networks, explainability and interpretability of the resulting models are much more challenging tasks that require the use of specifically developed methods to interpret the content of trained CNNs^36^.

### Stability of the results

In this section, the stability of the results obtained with 20 different random splits of the dataset into training/validation sets and testing set was evaluated. The results showed that the model using StandardScaling for normalization and SMOTENN to reduce the imbalance had better stability, with a deviation from the mean of 0.5%, 0.6% and 1.6% for training, validation, and testing, respectively (as shown in Table 9).

This could be due to the fact that StandardScaling normalizes the data to have a mean of 0 and a standard deviation of 1, which helps the model to learn more efficiently and reduces the risk of overfitting. Additionally, SMOTENN not only balances the dataset by oversampling the minority class but also cleans up the noise and borderline samples using the edited nearest neighbors algorithm. This makes the training data more representative and easier to learn.

However, the model that obtained the best classification results had slightly lower stability. This model used StandardScaling, ComBatOutlier method to harmonize the atypical data, and SMOTENN to balance the database. The average classification percentages for this model were 99.04%, 96.91%, and 93.4% for training, validation, and testing, respectively. The deviation from the mean was 0.9%, 1%, and 1.8% for training, validation, and testing, respectively.

This may be due to the fact that the ComBatOutlier method helps to adjust the batch effect and remove outliers, thus improving the quality of the data. However, this process may also introduce some instability, as the method is sensitive to the choice of reference sample and the amount of data available for harmonization. Despite this, the slight reduction in stability is acceptable given the substantial improvement in classification performance achieved by this model.

### Multicenter models

The second contribution presented in this paper concerns the potential improvement of the robustness of the models when applied to data of different origin. We have evaluated the performance of several models after a multicenter harmonization based on the criteria that allowed us to obtain the best results in our previous tests.

Multicenter models are essential when dealing with data from multiple sources or centers, as the data can have different characteristics and distributions. Therefore, it is necessary to harmonize the data to ensure that the models perform well on all datasets. In this study, we have performed multicenter harmonization using the ComBat method and StandardScaling normalization.

The comparison of the models (Table 13) highlighted that despite their good performance on their own database (87.55% and 93.26% respectively for IDC and BreaKHis), they do not perform well on data from the other database (58.16% and 47.20% respectively for IDC and BreaKHis). This demonstrates the clear need for an additional harmonization to improve the robustness of the model against external data.

The use of ComBat method for harmonization has been shown to be effective in previous studies. ComBat is a batch-effect correction method that adjusts for systematic variation between batches while preserving biological variability. It has been successfully applied to various biological datasets, including gene expression and neuroimaging data. In our study, we have used ComBat to adjust for any differences in the datasets that could affect model performance.

The best results obtained with multicenter harmonization were for the model that relied on StandardScaling method and outliers harmonization, with ComBat achieving 80% and 81% for the classification of patches from IDC and BreaKHis databases respectively. This result suggests that the use of both ComBat and StandardScaling can improve the performance of the models on external data. StandardScaling normalization scales the data to have zero mean and unit variance, making the data more comparable between different datasets. As with the best models presented above for IDC and BreaKHis, we provided additional details regarding the performance of our best multicenter models (Table 14). These models were trained to handle the multicenter problem, where the models were tested on patches from multiple datasets (IDC and BreaKHis).

### Discussion on our best model

**Figure 7:**
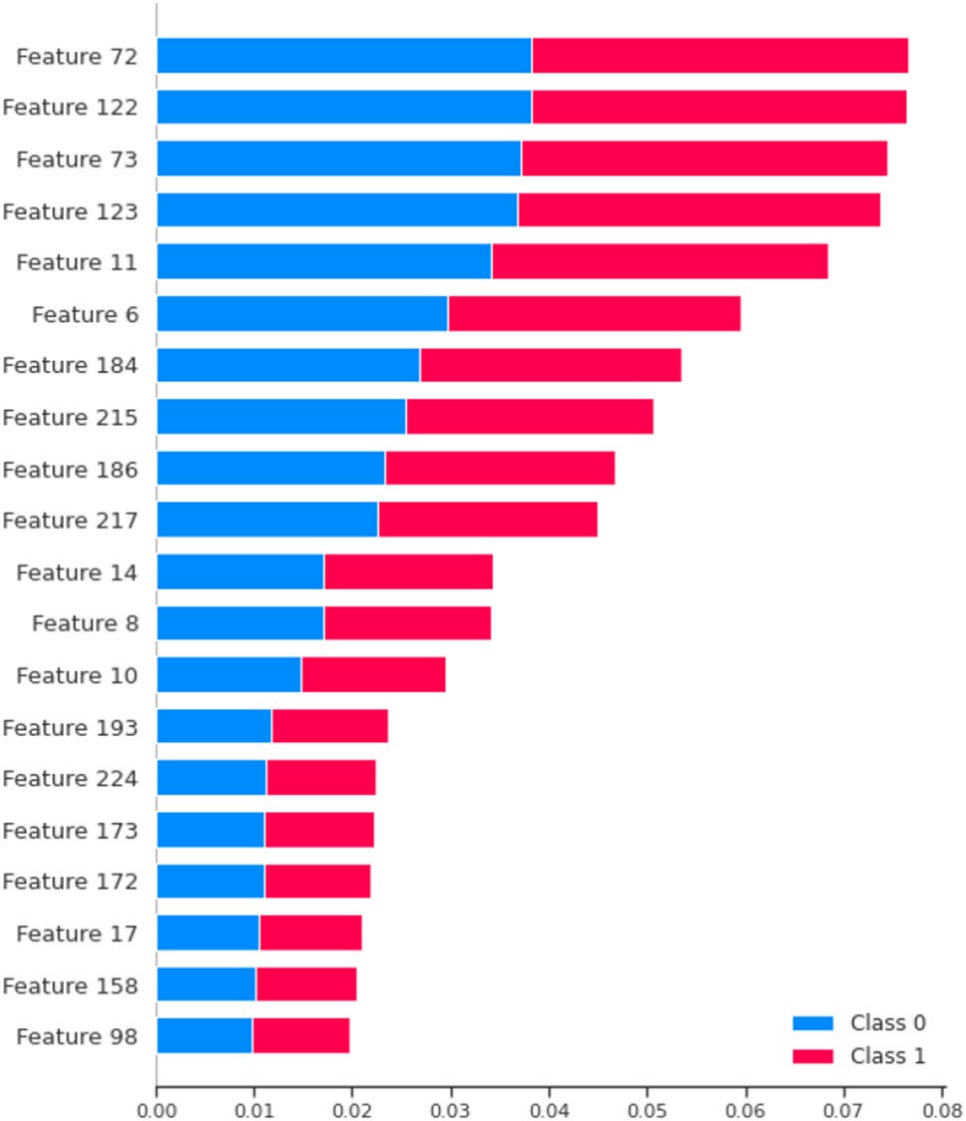
Results from the SHAP model on the IDCDB classification dataset, highlighting the most influential features for classification. Feature 72: Feat_Red_47; Feature 122 : Feat_Green_47; Feature 73 : Feat_Red_48; Feature 123 : Feat_Green_48; Feature 11 : longest_strike_above_mean; Feature 6 : autocorrelation; Feature 184 : Feat_Moments_Red_9; Feature 215 : Feat_Moments_Green_9; Feature 186 : Feat_Moments_Red_11; Feature 217: Feat_Moments_Green_11; Feature 14 : mean_change; Feature 8 : maximum; Feature 10 : kurtosis; Feature 193 : Feat_Moments_Red_18; Feature 224 : Feat_Moments_Green_18; Feature 173 : Feat_Blue_48; Feature 172 : Feat_Blue_47; Feature 17 : ratio_value_number_to_time_series_length; Feature 158 : Feat_Blue_33; Feature 98 : Feat_Green_23.

In our study, the optimal model configurations were identified as Configuration 6 (Standard Scaling, ComBat for Outlier Handling, and SMOTENN) for the IDC database, and Configuration 8 (Standard Scaling, ComBat for Outliers, SMOTENN, and Harmonisation by Patch) for the BreaKHis database. For BreaKHis, implementing Standard Scaling is pivotal to ensure all data attributes are on the same scale. The “ComBat for Outliers” method, as discussed in Section "Intra-database harmonization module", is crucial for aligning atypical samples to a reference cluster represented by non-atypical samples. Furthermore, the optimal data balancing method was determined to be SMOTENN, as elaborated in Section "Balancing". SMOTENN not only oversamples minority classes but also undersamples samples close to the majority class. The final pre-classification step involves Harmonization by Patch, which separates border patches from central patches and harmonizes them using the ComBat method, thereby reducing variability between these two clusters. For the IDC database, the same configuration settings, excluding the “Harmonisation by Patch” step, yielded the best results. The significance of the “Harmonisation by Patch” module becomes particularly evident when dealing with datasets that exhibit high intra-database variability and a limited number of samples. Such characteristics are prevalent in the BreaKHis dataset but are less pronounced in the IDC dataset. This specialized harmonization step helps in mitigating the adverse effects of variability within the database, thereby improving the model’s classification accuracy. The necessity for this step in BreaKHis, but not in IDC, could be attributed to the sufficiently large sample size in the IDC dataset (277524 patches), which allows the model to adequately learn despite the existing variability between central and border patches. Insights into feature importance are presented in Fig. 7 through the use of SHAP (Shapley Additive Explanations). We observe that the separation into RGB channels of the histopathological slides plays a significant role, with a higher number of influential features in the red (5 out of 20 features) and green (6 features) channels, as opposed to only 3 features from the blue channel, which had lesser influence on the classification. Additionally, general features like autocorrelation on pixel sequences were found to have a strong influence on the classification, implying a strong relationship between sequences of similar pixels. Regarding misclassifications, as demonstrated in Table 16, we found 3432 misclassified samples. Upon further analysis, Table 15 shows that 228 of these samples were misclassified with very low probability margins. Indeed, our model generates softmax probabilities for belonging to one of the two classes (IDC, non-IDC), and for these 228 samples, the classification deviated by only 1 to 5% from the true class label.

**Table 15:** 228 misclassified samples with minor prediction probability errors. Class 0 represents non-invasive ductal carcinoma, and Class 1 represents invasive ductal carcinoma. The table details the true labels, predicted labels, and the probabilities associated with each class, indicating a narrow discrepancy (1–5%) between the predicted and actual class labels.

Row_Num	True_Label	Predicted_Label	Probability_Class_0	Probability_Class_1
4	1	0	0.548590	0.451410
12	0	1	0.459256	0.540744
21	0	1	0.452394	0.547606
24	0	1	0.474211	0.525789
59	0	1	0.490471	0.509529
...	...	...	...	...
3382	1	0	0.538043	0.461957
3383	1	0	0.505659	0.494341
3385	1	0	0.548999	0.451001
3393	1	0	0.513401	0.486599
3401	1	0	0.507133	0.492867

**Table 16:** Summary of misclassified samples in the IDCDB dataset. Out of 277,524 samples obtained from 162 patients, a total of 3,432 samples were incorrectly classified by the model.

Row_Num	True_Label	Predicted_Label	Probability_Class_0	Probability_Class_1
0	1	0	0.595842	0.404158
1	1	0	0.991561	0.008439
2	0	1	0.135369	0.864631
3	1	0	0.966330	0.033670
4	1	0	0.548590	0.451410
...	...	...	...	...
3427	1	0	0.987699	0.012301
3428	0	1	0.005466	0.994534
3429	1	0	0.999739	0.000261
3430	0	1	0.013682	0.986318
3431	0	1	0.278023	0.721977

### Limitations

While our models have shown promising results, they also have several limitations that could be addressed in future iterations. One major drawback is the complexity of the model, which can make the classification process very time-consuming, particularly when dealing with large databases like IDCDB. To improve efficiency, we can explore various methods for harmonizing cluster patches, such as refining the harmonization process between patches and between patches by class. One possible solution is to export the coefficients of the ComBat algorithm used during the training phase, enabling us to harmonize the sample of test bases or even new data. Additionally, we can consider diversifying harmonization methods and incorporating numerical/clinical data to complement the images.

Another limitation lies in our feature engineering approach, which while successful in producing an accurate interpretation of relevant features that influence the results (approximately 94%), may be inadequate in representing the input images. To address this, we can integrate the weight matrices from the convolution, pooling, and activation layers in the initial architecture of a deep learning network with the computed features. This will provide a better representation of the inputs and potentially improve the classification performance. It is worth noting that deep learning approaches have achieved similar or even better results on the BreaKHis Dataset for breast cancer, as reported in the literature^31,33,37,38^.

## Conclusions

The ComBat method is currently well known for feature realignment from multicenter data, but we introduce ComBat with automatic outlier detection to address non-biological variability, such as acquisition defects and internal edge staining in the case of histopathology slides. We also worked on different applications of the ComBat method within the same database to present two other harmonization approaches: Patch Harmonization and Patch Harmonization in each class. We noticed that for the BreaKHis dataset, harmonizing ComBatOutliers is sufficient to obtain results that rival the literature, however, with the IDC dataset, applying the patch harmonization approach with ComBatOutliers provides the best results. This may be due to the volume of the dataset where a massive dataset like IDC (277524 patches) contains much more variability than BreaKHIs (1490 patches). The pipeline contains different blocks that will allow the user to adapt the use of the harmonizations according to his study case.

In this paper we presented two contributions dedicated to improve the performance of classification in the presence of heterogeneous data, including different databases, in the context of benign/cancerous classification of patches from histological slides. Our first contribution was a pipeline made available to researchers working in this field. It is implemented here for the classification of malignant and benign patches in invasive ductal carcinoma of the breast. This pipeline has shown effectiveness in producing models that outperformed recent ones from the literature based on feature engineering. The second contribution concerns the harmonization of data at different stages of the process, both to deal with the problem of outliers and to resolve the pitfalls of multicenter studies. This hybrid harmonization shows its robustness by comparing the results with our best models without harmonization. This robustness concerns the classification of images coming from different acquisition sources, with practically identical efficiency on the two databases used in this work. The proposed pipeline will now be evaluated in different applications and contexts, such as FDG positron emission tomography based radiomics predictive models in lymphoma.

## Data Availability

The datasets used in this study were obtained from two open-access sources. The BreaKHis dataset^13^ was acquired by requesting access from the authors. The Invasive Ductal Carcinoma (IDC) dataset, described and published in Ref.^12^, is available on Kaggle at the following link: https://www.kaggle.com/datasets/paultimothymooney/breast-histopathology-images.
